# T-Cell Memory Responses Elicited by Yellow Fever Vaccine are Targeted to Overlapping Epitopes Containing Multiple HLA-I and -II Binding Motifs

**DOI:** 10.1371/journal.pntd.0001938

**Published:** 2013-01-31

**Authors:** Andréa Barbosa de Melo, Eduardo J. M. Nascimento, Ulisses Braga-Neto, Rafael Dhalia, Ana Maria Silva, Mathias Oelke, Jonathan P. Schneck, John Sidney, Alessandro Sette, Silvia M. L. Montenegro, Ernesto T. A. Marques

**Affiliations:** 1 Virology and Experimental Therapeutics Laboratory, Aggeu Magalhães Research Center, Fiocruz, Recife, Pernambuco, Brazil; 2 Center for Vaccine Research, Department of Infectious Diseases and Microbiology, University of Pittsburgh, Pittsburgh, Pennsylvania, United States of America; 3 Department of Electrical and Computer Engineering, Texas A&M University, College Station, Texas, United States of America; 4 Department of Pathology, The Johns Hopkins University School of Medicine, Baltimore, Maryland, United States of America; 5 La Jolla Institute for Allergy and Immunology, Vaccine Discovery, La Jolla, California, United States of America; 6 Department of Immunology, Aggeu Magalhães Research Center, Fiocruz, Recife, Pernambuco, Brazil; University of Rhode Island, United States of America

## Abstract

The yellow fever vaccines (YF-17D-204 and 17DD) are considered to be among the safest vaccines and the presence of neutralizing antibodies is correlated with protection, although other immune effector mechanisms are known to be involved. T-cell responses are known to play an important role modulating antibody production and the killing of infected cells. However, little is known about the repertoire of T-cell responses elicited by the YF-17DD vaccine in humans. In this report, a library of 653 partially overlapping 15-mer peptides covering the envelope (Env) and nonstructural (NS) proteins 1 to 5 of the vaccine was utilized to perform a comprehensive analysis of the virus-specific CD4^+^ and CD8^+^ T-cell responses. The T-cell responses were screened *ex-vivo* by IFN-γ ELISPOT assays using blood samples from 220 YF-17DD vaccinees collected two months to four years after immunization. Each peptide was tested in 75 to 208 separate individuals of the cohort. The screening identified sixteen immunodominant antigens that elicited activation of circulating memory T-cells in 10% to 33% of the individuals. Biochemical *in-vitro* binding assays and immunogenetic and immunogenicity studies indicated that each of the sixteen immunogenic 15-mer peptides contained two or more partially overlapping epitopes that could bind with high affinity to molecules of different HLAs. The prevalence of the immunogenicity of a peptide in the cohort was correlated with the diversity of HLA-II alleles that they could bind. These findings suggest that overlapping of HLA binding motifs within a peptide enhances its T-cell immunogenicity and the prevalence of the response in the population. In summary, the results suggests that in addition to factors of the innate immunity, “promiscuous” T-cell antigens might contribute to the high efficacy of the yellow fever vaccines.

## Author Summary

T-cell responses are considered to be very important; however, the role of T-cell responses in vaccine mediated immunity is still controversial. One reason may be that most studies of human T-cell responses are focused on a few epitopes. We still lack a systematic view of the repertoire of peptides presented by the different HLA class I and II molecules and how the peptides presented by the different HLAs interact within the host to develop T-cell responses. Here we present a study of the T-cell responses against the YF-17DD vaccine in the context of a cohort of 220 volunteers and observed that the most prevalent T-cell responses are targeted at peptides that bind to multiple types of HLA molecules. Based on these results we postulate that promiscuous T-cell epitopes might have a critical role in the development of adaptive immunity. These results may have broader implications for other pathogens, since the yellow fever vaccine is currently being developed as a vaccine vector for other diseases. Therefore, these epitopes might have a functionally cooperative role in boosting specific neutralizing antibody responses. In addition, we propose that promiscuous T-cell antigens may be better immunogens for vaccine development; however more studies are necessary.

## Introduction

The yellow fever (YF) vaccines (YF-17D-204 and 17DD) are considered to be among the most effective vaccines [1], [2]. Antibody and T-cell responses are believed to mediate protection [3], [4], [5], and recent studies have also implicated the innate immune responses as one of the critical elements for developing the immune responses [6]. However, the immune adaptive mechanisms that make this vaccine so highly effective are unclear. T-cell immune responses against YF wild type virus and other flaviviruses, such as dengue and West Nile virus [7], [8], are considered to be important for development of neutralizing antibodies, and activation of CD4^+^ helper T-cells and CD8^+^ cytotoxic T lymphocytes (CTLs) against YF wild type virus has been reported [6], [9], [10]. The CTL responses appear 14 days after vaccination and these cells differentiate into long-lived memory T-cells after a few months [11]; however, only a few YF wild type virus T-cell epitopes have been described in humans [12], [13].

In order to expand the repertoire of human leukocyte antigens (HLA) restricted YF wild type virus epitopes, and as part of the Immune Epitope Database - IEDB program (http://www.immuneepitope.org/), we studied the repertoire of T-cell responses present in a cohort of YF-17DD vaccinees established by Melo et al. [14] and identified 16 peptides that are immunogenic in more than 10% of the individuals tested. Analysis of the most prevalent immunogenic peptides indicated that they contain overlapping HLA binding motifs and suggested that the prevalence T-cell immunogenicity in response to YF vaccine (17DD) is correlated with the ability of the peptide to bind multiple HLA types.

## Materials and Methods

### Ethics statement

This study was reviewed and approved by the ethics committee of the Brazilian Ministry of Health (CONEP: 12138; Process n° 25000.103608/2005-39). In addition, the Johns Hopkins University and University of Pittsburgh Institutional Review Boards also reviewed and approved this study as protocol JHM-IRB-3: 03-08-27-01 and PRO09090146 respectively. Written informed consent was obtained from all volunteers and all clinical investigation was conducted according to the principles expressed in the Declaration of Helsinki.

### Study Population

A cohort of YF-17DD vaccinees was established and described elsewhere [14]. Briefly, healthy subjects, IgG-negative for YFV, were immunized with the YF-17DD vaccine (Biomanguinhos, Oswaldo Cruz Foundation, Brazil) at The Brazilian National Health Surveillance Agency (ANVISA) office, International Airport of Recife. After a full explanation of the study, written consent was obtained from each volunteer and blood samples were collected before (Day 0, used as negative controls) and after vaccination (2 months to 4 years).

### Blood Collection and Isolation of Peripheral Blood Mononuclear Cells (PBMCs)

Blood samples were collected from 220 subjects into 10 mL Vacutainer® tubes (Becton Dickinson, Franklin Lakes, NJ). Serum was obtained by centrifuging the whole blood at 1,600×g for 10 min and aliquots (1 mL per tube) were stored at −20°C for further use on serological tests. For harvesting PBMC, blood samples collected in heparin Vacutainer® tubes (Becton Dickinson, Franklin Lakes, NJ) were used. Isolation of PBMC was carried out through gradient density using Ficoll-Paque™ PLUS (Amersham Biosciences Ab, Uppsala, Sweden) according to the protocol suggested by the manufacturer. Residual red blood cells were lyzed with Ammonium-Chloride-Potassium lysing buffer (Gibco BRL, Gaithersburg, MD) for 3 min at room temperature. Then, cells were resuspended in Hybridoma Serum Free Media (SFM; Gibco BRL, Gaithersburg, MD) for ELISPOT assaying.

### CD4^+^ T-Cell Depletion

PBMC samples from at least 3 subjects, known to respond against the peptides Env_57–71_, Env_345–359_, Env_361–375_, NS3_137–151_, NS4b_77–91_, NS5_341–355_, NS5_345–359_, NS5_465–479_ and NS5_481–495_ were depleted of CD4 T-cells using anti-CD4 mAb coated microbeads (Miltenyi Biotec, Auburn, CA) and LD columns (Miltenyi Biotec, Auburn, CA) according to manufacturer's directions. This approach consistently yielded cell samples in which depletion was greater than 95% for CD4^+^ T-cells, according to flow cytometry analysis (data not shown).

### Isolation of CD8^+^ T-Cells

CD8^+^ T-cells were isolated from PBMC samples by the negative selection method using the isolation kits and LS columns provided by Miltenyi Biotec (Auburn, CA), according to the manufacturer's manual. This approach consistently yielded cell samples in which purity was greater than 90% for CD8^+^ T-cells according to flow cytometer analysis (data not shown).

### Peptide Library

To measure the breadth and magnitude of T-cell responses, a library of 653 peptides (15-mers), overlapping by 11 amino acids, spanning the length of the 17DD YFV proteins Env (n = 120), NS1 (n = 47), NS2a (n = 32), NS2b (n = 28), NS3 (n = 148), NS4a (n = 45), NS4b (n = 17) and NS5 (n = 216) (sequence NCBI entry U17066) was synthesized by Schafer-N (Copenhagen, Denmark). These peptides were HPLC-purified to 80% purity or greater, with the exception of four peptides that could not be purified and were used as crude products.

The Env peptides were pooled in groups of 6 peptides, totalizing 20 pools, Box S1. NS peptides were arranged into matrix pools [NS1 (7×7); NS2a (8×4); NS2b (7×4); NS3 (15×10); NS4a (8×5); NS4b (4×4) and NS5 (17×13)] ensuring that each peptide was present in two different pools, Box S2 [15]. Ten NS peptides were tested individually (no pooling).

The peptides NS4b_77–85_, NS4b_76–84_, NS4b_75–83_, NS4b_75–84_ were synthesized and purified by Genescript (New Jersey, USA). The purity of these peptides was greater than 95%.

Stock solutions of all peptides were sterile-prepared at 2 mg/mL in 10% (v/v) dimethil sulphoxide (DMSO; Sigma-Aldrich) and stored at −20°C until use.

### Cell Lines and Peptide Pulsing

Transporter associated with antigen processing-deficient T2 cells expressing HLA-A*0201 were maintained in RPMI 1640 medium (Invitrogen, Brown Dee, WI) supplemented with 10% (v/v), fetal bovine serum (FBS; Hyclone, Logan, UT), 1% (v/v) glutamine (Gibco, Gaithersburg, MD) and 1% (v/v) penicillin-streptomycin (Gibco, Gaithersburg, MD). For pulsing the peptides, one million target cells were washed, resuspended in 1 mL of media and incubated at 37°C for 2 h with peptides at 10 µg/mL. Cells were then washed 3 times and resuspended in SFM at 1×10^6^ cells/mL for T-cell function assays.

### ELISPOT Assay for IFN-γ

Interferon-γ ELISPOT assays were performed by using the ELISPOT set from BD-Biosciences Pharmingen (San Diego, CA), according to the manufacturer′s protocol. Briefly, 96-well nitrocellulose-bottom plates were coated with 100 µL/well primary antibody anti-human IFN-γ at 5 µg/mL and incubated at 4°C overnight. The following day, plates were blocked with 200 µL/well of RPMI 1640 (Invitrogen, Brown Dee, WI) containing 10% (v/v) heat-inactivated FBS (Hyclone, Logan, UT), 1% (v/v) glutamine (Gibco, Gaithersburg, MD) and 1% (v/v) penicillin-streptomycin (Gibco, Gaithersburg, MD) for 2 h at room temperature. Then, the cells were plated, in duplicate, at a range of 1–3×10^5^ cells/well along with peptides at 10 µg/mL (concentration of each peptide when tested as a pool and also the concentration of individual peptides when tested individually) both in SFM. During peptide pool analysis and deconvolution, total PBMC were used as source of T-cells. For the experiments aimed to confirm whether CD8^+^ T-cells were driving the cellular responses, in addition to total PBMC, CD4-depleted PBMC samples were included in the analysis. Media (SFM) containing 1% (v/v) glutamine, 1% (v/v) penicillin-streptomycin alone (background) and media supplemented with phorbol 12-myristate 13-acetate (PMA; Sigma) at 250 ng/mL and ionomycin (Sigma) at 250 ng/mL were used as negative and positive controls, respectively. After 16 h incubation at 37°C, 5% CO_2_, the plates were washed twice with distilled water and 4 times with PBS containing 0.05% Tween-20 (PBS-T; Sigma) followed by incubation for 2 h, at room temperature, with 4 µg/mL of biotinylated anti-human IFN-γ. Plates were again washed four times with PBS-T and incubated for 1 h, at room temperature, with avidin-horseradish peroxidase at 10 µg/mL. After another washing cycle (5×) using PBS-T followed by three washes with PBS, plates were developed with substrate 3-amino-9-ethyl carbazole (AEC; BD-Biosciences Pharmingen, San Diego, CA) for 40 min. The reaction was stopped with distilled water, the plates were air-dried and spots were counted using the ImmunoSPOT reader using ImmunoSPOT software version 3.2 (Cellular Technology Ltd.). Totals for duplicated wells were averaged and normalized to numbers of IFN-γ spot forming cells (SFC) per 1×10^6^ PBMCs.

In experiments aimed at confirm the HLA-A*0201 restriction of T-cell epitopes identified, ELISPOT was performed using T2 cell lines as target to activate CD8^+^ T-cells. T2 cells were pulsed with each of the NS4b peptides (NS4b_77–85_, NS4b_76–84_, NS4b_75–83_ and NS4b_75–84_) individually. Peptide-pulsed target cells and effector cells (CD8 T-cells) were co-cultured at 1×10^5^ cells/well each and incubated for 16 h at 37°C, 5% CO_2_ on ELISPOT plate. After incubation, the cells were removed and IFN-γ was detected as described above. The negative controls were effector cells only and effector cells plated with unpulsed target cells, whereas the positive control was PMA/ionomycin as mentioned previously.

The criteria to identify positive peptide pools/individual peptides relied on the combination of the following equations: (i) mean number of spots (peptides)−2 standard deviations (SD)>mean number of spots (background); (ii) mean number of spots (peptides)>mean number of spots (background)+2 SD; (iii) mean number of spots (peptides) - mean number of spots (background)>10. The peptide pools that met all criteria listed above were directly selected. For the NS proteins, T-cell responses among volunteers analyzed during pool analysis was low and, thus, difficult to comply with the analysis criteria shown above; therefore, an additional test was applied in order to increase the number of pools to deconvolute. This additional test consisted of the calculation of a *cut-off* based on average (Avg) SFC of the top 25% peptide pool and standard error through the following equation: *Avg SFC Top 25% peptide pool +2 SE*. The peptide pool in which SFC value was greater than the cut-off was also considered positive. The combinations in which the frequency was greater than 7% (for NS1 and NS3 peptides); 10% (for NS2a, NS2b, NS4a and NS4b); and 16% to NS5 were taken into account, and their respective common peptide was selected to be tested individually.

### HLA Genotyping

Genomic DNA was extracted from PBMCs collected from 142 volunteers by using the PureLink Genomic DNA MiniKit (Invitrogen, Carlsbad, CA) following the manufacturer protocol. HLA alleles genotyping was performed using the polymerase chain reaction (PCR) test sequence-specific primer (SSP) UniTray Kit (Invitrogen, Brown Dee, WI), which provides low to intermediate HLA typing results according to the manufacturer protocol. After amplification, the PCR products were separated in 2% (w/v) agarose gel pre-stained with ethidium bromide (0.25 µg/mL gel). Gels were electrophoresed for 30 min at 150 volts in 0.5× Tris-Boric acid-EDTA (TBE) buffer, then examined under Ultraviolet illumination and documented by photography. The types of HLA-A, B, C were then determined by specific electrophoresis bands using the UniMatch Software (Invitrogen, Brown Dee, WI).

Further analysis was performed to determine the HLA allele in high-resolution for the individuals HLA-A*02 positives using the same methodology above mentioned.

### Artificial APC (aAPC) Loading and Expansion of NS4b-Specific CD8 T-Cells

The peptides NS4b_77–85_, NS4b_76–84_, NS4b_75–83_ and NS4b_75–84_ were incubated individually with HLA-A*0201/anti-hCD28 aAPCs [16], [17], [18] at concentration of 50×10^6^ beads/mL for 5 days at 4°C. Then, one million CD8^+^ T-cells isolated from HLA-A*0201 positive individuals were co-cultured with 1×10^6^ of each peptide-loaded aAPCs, individually, in RPMI 1640 media supplemented with 8% (v/v) T-cell growth factor (TCGF) and 5% (v/v) human AB serum. On day 4, the plates were replenished with media containing TCGF and AB human serum and cultured for 3 more days. On day 7, the aAPCs were removed and the cells were washed, counted and their concentration adjusted to 1×10^6^ cells/mL in RPMI 1640 media (Invitrogen, Brown Dee, WI) supplemented with 10% (v/v) FBS (Hyclone, Logan, UT).

### Intracellular Cytokine Staining (ICS) to Detect NS4b-Specific CD8^+^ T-Cell Responses

CD8^+^ T-cells (1×10^5^) that underwent one round of expansion with different aAPCs were “challenged” with T2 cells (1×10^5^) pulsed, individually, with each NS4b peptides for 2 h at 37C, 5% CO_2_. Then, Golgi stop (BD Biosciences, San Diego, CA) was added to the culture to stop protein trafficking and secretion. The cells were cultured for 4 h at 37C, 5% CO_2_ and then washed twice with staining buffer [PBS containing 0.05% sodium azide and 2% (v/v) FBS (Hyclone, Logan, UT)]. The cells were stained with anti-CD8 FITC (Sigma Aldrich, St. Louis, MO) for 20 min at 4°C and washed 3 times with staining buffer. After that, the cells were permeabilized with cytoperm/cytofix buffer (BD Biosciences, San Diego, CA) for 20 min at 4°C followed by 3 washes with perm/wash buffer (BD Biosciences, San Diego, CA). Then staining with either anti-hCD107a-PE (BD Biosciences, San Diego, CA) or anti-hIFN-γ-PE (BD Biosciences, San Diego, CA) was performed for 30 min at 4°C, after which the cells were washed twice with perm/wash buffer and once with staining buffer. Finally, the cells were acquired on a FACS Calibur flow cytometer (BD Bioscience, San Diego, CA) and the data was analyzed using Flowjo for Macintosh version 8.8.6 (Tree star, Ashland, OR).

### MHC Purification and Binding Assays

Purification of HLA class II MHC molecules by affinity chromatography, and the performance of assays based on the inhibition of binding of a high affinity radiolabeled peptide to quantitatively measure peptide binding, were performed essentially as detailed elsewhere [19], [20], [21], [22], [23]. Briefly, EBV transformed homozygous cell lines were used as sources of MHC molecules. A high affinity radiolabeled peptide (0.1–1 nM) was co-incubated at room temperature or 37C with purified MHC in the presence of a cocktail of protease inhibitors. Following a two-day incubation, MHC bound radioactivity was determined by capturing MHC/peptide complexes on Ab coated Lumitrac 600 plates (Greiner Bio-one, Frickenhausen, Germany), and measuring bound cpm using the TopCount (Packard Instrument Co., Meriden, CT) microscintillation counter. The concentration of peptide yielding 50% inhibition of the binding of the radiolabeled peptide was calculated. Under the conditions utilized, where [label]<[MHC] and IC50≥[MHC], the measured IC50 values are reasonable approximations of the true Kd values. Each competitor peptide was tested at six different concentrations covering a 100,000-fold range, and in three or more independent experiments. As a positive control, the unlabeled version of the radiolabeled probe was also tested in each experiment.

### Statistical Analysis

Fisher's two-tailed test was used to associate the response against a given peptide and HLA genotype. When significant p-values were achieved (p<0.05), the odds ratio and 95% confidence intervals (CI) were calculated (R Statistical Package 2.9.0). HLA-A, B and C genotype distributions were checked with Hardy-Weinberg equilibrium. In addition, we compared the allelic distributions of the five genotyped HLA genes (HLA-A, HLA-B, HLA-C, HLA-DR, HLA-DQ) to HLA genotype data obtained from the MHC Database (dbMHC) of the NCBI, consisting of two studies with Brazilian populations: one with volunteers from the state of Minas Gerais (which we refer to as “dbMHC1”), and another with volunteers from the state of São Paulo (referred to as “dbMHC2”). Pearson's correlation was used to assess the co-variation between HLA promiscuity and frequency of recognition of the yellow fever T-cell epitopes identified.

## Results

### Screening of Immunogenic Peptides

**Figure 1:**
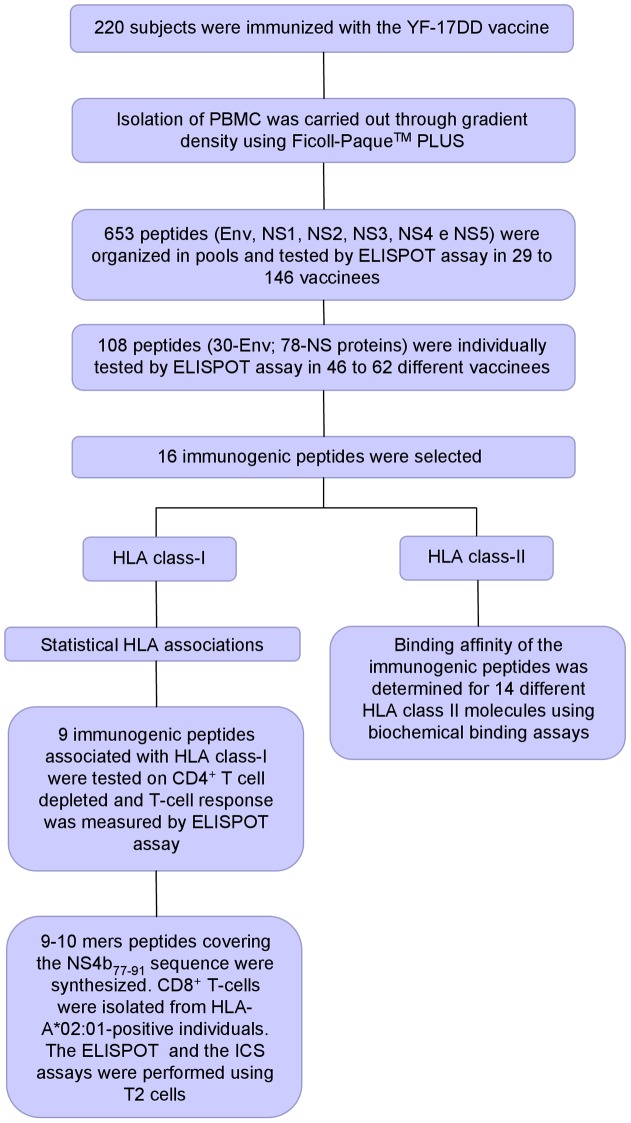
Screening of immunogenic yellow fever peptides. Blood samples were obtained from 220 vaccinees. 653 peptides from Env and non-structural proteins were organized in pools and tested by ELISPOT assay. Then, 108 immunogenic peptides were selected from the pools and tested individually. In total, 16 peptides were positive in at least 10% the subjects. Statistical association showed that nine immunogenic peptides were associated with HLA class-I. Seven from these peptides could activate CD4^+^ depleted PBMCs from YF-17DD vaccinees *ex vivo*. In addition, 9–10 mers peptides covering the NS4b_77–91_ were cultured in CD8^+^ T-cells isolated from HLA-A*02:01-positive individuals. Four epitopes (NS4b_77–85_, NS4b_75–83_, NS4b_75–84_, NS4b_76–84_) were able to induce a specific response. Biochemical binding assays indicated that the most prevalent immunogenic peptides could bind multiple HLA-II molecules.

The immunogenicity of the YF-17DD peptides was determined by IFN-γ ELISPOT performed *ex vivo* using PBMCs from immunized volunteers. A flowchart summarizing the assays and strategies for characterization of the T-cell responses are show in Figure 1. Blood samples were negative for anti-YF-17DD antibodies prior to vaccine inoculation and all vaccinees seroconverted by one month after immunization [14]. The peptides were organized in groups corresponding to the Env, NS1, NS2, NS3, NS4 and NS5 proteins and screened in two rounds. In the first round the peptides were organized in pools or matrices and tested in samples from a series of donors as indicated in Table 1. In the second round, immunogenic peptides were selected from the pools screened individually as indicated in Table 2.

**Table 1:** Summary of screening peptide pools (first round).

Protein	No. of peptides	No. vaccinees	No. of peptides recognized
Env	120	146	20*
NS1	47	29	30
NS2a	32	52	27
NS2b	28	52	25
NS3	148	29	61
NS4a	45	52	38
NS4b	17	52	17
NS5	216	37	192

The table shows a list of all yellow fever proteins mapped, total number of peptides tested for each protein (No. of peptides), volunteers (No. vaccinees) and peptides into a pool that were positive at least one subject (No. of peptides recognized).

* Env peptide pools. Twenty peptide pools were positive at least one subject.

**Table 2:** Summary of screening individual peptides (second round).

Protein	No. of peptides	No. vaccinees	No. of peptides recognized
Env*	30	62	13
NS1	9	46	7
NS2a	3	59	3
NS2b	6	59	6
NS3	18	46	18
NS4a	17	59	11
NS4b	4	59	2
NS5	21	48	13

The table shows a list of all yellow fever proteins mapped, total number of individual peptides tested (No. of peptides), volunteers (No. vaccinees) and individual peptides that were positive at least one subject (No. of peptides recognized).

* The most immunogenic Env pools (3, 9, 14, 15 e 20) were selected and thirty peptides (six peptides in each pool) were tested individually.

Screening of immunogenic T-cell peptides within the structural proteins: The initial screening was performed with 120 peptides from the Env protein. The peptides were organized in 20 pools with six peptides in each and tested in samples from 146 volunteers. Five pools (3, 9, 14, 15 and 20) were immunogenic in 13 to 34 of the 146 individuals tested. Peptide pool number 15 was immunogenic in 34 of the 146 individuals tested and was most prevalent immunogenic pool in this population (data not shown). Thirty individual peptides from these five pools were selected for a second round of analysis and tested individually (Table 2). Thirteen peptides were positive in the second round (Table S1). Six (Env_57–71_, Env_65–79_, Env_73–87_, Env_337–351_, Env_345–359_ and Env_361–375_) out of the 30 Env peptides tested individually were positive in at least 10% the volunteers. Their protein position, amino acid sequence, frequency with in the cohort and magnitude of the *ex vivo* T-cell response are shown on Table 3.

**Table 3:** Most frequent peptides that activated T-cells from YF-17DD vaccinees.

Peptide	Sequence	(n) 2^nd^ round	No. of responders	Recognition frequency (%)	Average ± STDEV	Median (peptide)	Median (negative control)
Env_57–71_	RKVCYNAVLTHVKIN	30	10	33	149±248	48	2
Env_65–79_	LTHVKINDKCPSTGE	30	3	10	158±196	83	5
Env_73–87_	KCPSTGEAHLAEENE	30	3	10	146±153	60	2
Env_337–351_	ADDLTAAINKGILVT	18	3	17	23±10	28	3
Env_345–359_	NKGILVTVNPIASTN	27	9	33	72±85	30	3
Env_361–375_	DEVLIEVNPPFGDSY	27	4	15	72±88	35	9
NS2b_97–111_	VVMTSLALVGAALHP	59	6	10	14±6	12	4
NS2b_113–127_	ALLLVLAGWLFHVRG	22	3	14	80±89	43	2
NS3_137–151_	GTSGSPIVNRNGEVI	46	5	11	90±49	115	5
NS4a_197–211_	TMLSPMLHHWIKVEY	22	3	14	140±178	61	3
NS4b_77–91_	LWNGPMAVSMTGVMR	59	11	19	100±138	50	3
NS5_341–355_	RMAMTDTTPFGQQRV	48	6	12	92±93	52	5
NS5_345–359_	TDTTPFGQQRVFKEK	48	8	17	336±320	172	3
NS5_465–479_	EFGKAKGSRAIWYMW	48	6	12	486±707	67	2
NS5_469–483_	AKGSRAIWYMWLGAR	48	8	17	395±643	45	3
NS5_481–495_	GARYLEFEALGFLNE	48	11	23	383±380	204	2

(n) = Number of volunteers tested against each individual peptide.

Screening of immunogenic T-cell peptides within Non-structural (NS) proteins. A similar strategy was applied to screen the T-cell responses against NS peptides. In the first round, the pools were organized in matrices with each peptide being present in two pools to allow identification of the immunogenic peptide within each pool. The matrices were assembled as described in the methods section and tested in a series of samples from different volunteers (Table 1). The peptide pool matrix analysis, in many cases, allowed the precise identification of the immunogenic peptide within each pool. The peptides that most frequently activated T-cell responses in the individuals tested are shown in Table S1. These peptides were selected for the second round of screening as individual peptides in a second set of samples. The summary of the results of the second round is shown in Table 2. In total, 7 (NS1), 3 (NS2a), 6 (NS2b), 18 (NS3), 11 (NS4a), 2 (NS4b) and 13 (NS5) peptides were shown to be immunogenic on 1 to 11 vaccinees tested in the second round (Table S1). Ten peptides among NS proteins (NS2b_97–111_, NS2b_113–127_, NS3_137–151_, NS4a_197–211_, NS4b_77–91_, NS5_341–355_, NS5_345–359_, NS5_465–479_, NS5_469–483_, NS5_481–495_) were immunogenic in at least 10% of the YF-17DD vaccinees. Their position, sequence, frequency and magnitude of T-cell response are shown on Table 3.

### Identification of HLA Class I Alleles Associated with the Most Frequently Immunogenic Peptides

In order to investigate the HLA restriction related to the T-cell responses to each of the peptides shown on Table 3, HLA genotyping was performed for the loci HLA-A, HLA-B and HLA-C on 142 YF-17DD vaccinees (Table S2). The frequency of the HLA types present in our cohort was compared to the frequencies reported by two Brazilian blood banks (Minas Gerais - “dbMHC1” and São Paulo - “dbMHC2”) and deposited at the cohort dbMHC database. Statistical analysis indicated a large degree of correlation between the HLA diversity present in the study volunteers and the ones on the dbMHC database, for all available genes (p<0.01), suggesting that the HLA diversity of the cohort is representative of the general Brazilian population. The HLA types of the samples used on the second round of screening and the respective peptide with which they reacted are shown in Table S3. Subsequently, we investigated the possibility of some HLA types to be overrepresented among the individuals responding to a peptide. Indeed, some HLA types were very prevalent among the responders of some peptides. For example, HLA-A*23 was very frequent among the individuals responding to the NS5_481–495_ peptide while it was seldom observed among individuals that did not react to this peptide suggesting the possibility that this HLA type might be involved in the presentation of an epitope present within the immunogenic 15-mer. In order to confirm these associations, Fisher's test was carried out comparing the frequency of the HLA types present in the vaccinees responding to a given peptides versus the HLA frequency in the general population or among the ones that did not respond. The HLA alleles with significant statistical associations are shown in Table 4.

**Table 4:** Statistical HLA class I association analysis.

Peptide	HLA Association
Env_57–71_	A*02 (OR = 3; p = 0.03)
Env_345–359_	A*02 (OR = 5; p = 0.003)
Env_361–375_	A*26 (OR = 9; p = 0.03)
	B*18 (OR = 10; p = 0.01)
NS3_137–151_	A*11 (OR = 3; p = 0.03)
NS4b_77–91_	A*02 (OR = 3; p = 0.02)
NS5_341–355_	B*39 (OR = 13; p = 0.004)
	C*12 (OR = 8; p = 0.006)
NS5_345–359_	B*39 (OR = 9; p = 0.01)
NS5_465–479_	B*15 (OR = 5; p = 0.04)
NS5_481–495_	A*23 OR = 6; p = 0.001

Nine out of the 16 immunogenic peptides could be statistically associated with one or more HLA type. Seven peptides presented significant associations with one HLA type, four HLA-A (A*02, A*11, A*23, A*26), three HLA-B (B*15, B*18, B*39) and one HLA-C (C*12). Two peptides, Env_361–375_ and NS5_341–355_ showed significant association with two HLA-types (A*26 and B*18) and (B*39 and C*12) respectively. Three immunogenic peptides Env_57–71_, Env_345–359_ and NS4b_77–91_, were associated with HLA-A*02 and other two NS5_341–355_ and NS5_345–359_ were associated with B*39. However these two peptides associated with B*39 overlap by 11 amino acids suggesting that these associations are likely directed to an immunogenic determinants shared by these two adjacent peptides.

### Validation of HLA Class I Association Strategy and Confirmation of HLA-A*02 CD8^+^ T-Cell-Restricted Responses

The peptides Env_57–71_, Env_345–359_ and NS4b_77–91_ induced immune responses predominantly in HLA-A*02 individuals (Tables 4 and S3). In addition, the statistical analysis indicated that the peptides Env_361–375_, NS3_137–151_, NS5_341–355_, NS5_345–359_, NS5_465–479_ and NS5_481–495_ were associated with the presence of the other HLA class I alleles. In order to validate these associations, we analyzed whether CD8^+^ T-cells derived from the immunized individuals could be activated by these peptides. For these analysis, PBMCs from 3 volunteers were collected, the CD4^+^ T-cell depleted, and the T-cell responses to each peptide measured by ELISPOT. It is important to note that some antigen presenting cells (APC) were unintentionally removed, because CD4 is also expressed on monocytes and dendritic cells, albeit at lower levels than on CD4^+^ T helper cells. Seven of the nine peptides selected could activate CD4^+^ depleted PBMCs from YF-17DD vaccinees *ex vivo*, whereas PBMCs collected pre-vaccination were negative, corroborating the hypothesis that these immune responses are likely being mediated by CD8^+^ T-cells (Table 5).

**Table 5:** Determination of memory CD8 T-cell-restricted responses against the peptides associated with HLA class I molecules.

Peptide	No. of responders	IFN-γ SFC (CD4-depleted PBMCs/10^6^)	
		Average ± STDEV	Median
Env_57–71_	3	152±145	84
Env_345–359_	3	315±160	287
Env_361–375_	2	16±0	16
NS3_137–151_	0	-	-
NS4b_77–91_	3	101±23	101
NS5_341–355_	0	-	-
NS5_345–359_	3	66±71	32
NS5_465–479_	2	168±0	168
NS5_481–495_	2	95±0	95

(−) = No response.

**Figure 2:**
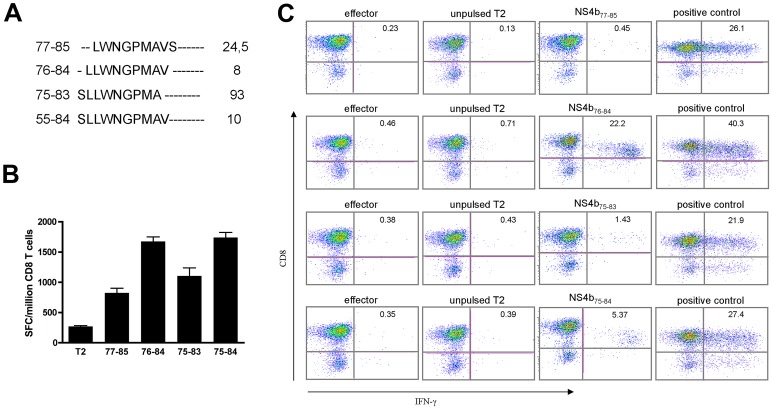
YF-17DD Immunodominant HLA-A*0201 restricted epitope in the NS4b protein. **A**. Sequences of the peptides used to identify the immunogenic nonamer epitope in NS4b. IC_50_ values for IEDB-AR prediction were calculated; **B**. PBMCs from HLA-A2 vaccinees were cultured in the presence or absence of 9–10mer (NS4b_77–85_, NS4b_76–84_, NS4b_75–83_, NS4b_75–84_) peptides. ELISPOT assay; **C.** Intracellular cytokine staining were performed. All peptides analyzed could activate CD8^+^ T-cells and the expansion of NS4b_76–84_-specific CD8^+^ T-cells was the highest among the four peptides tested. Plots gated on CD8^+^ T-cells for one representative donor are shown.

In order to further determine the HLA-restriction involved on the T cell response against the peptide NS4b_77–91_, previously shown to be associated with HLA-A*02, all the subjects bearing this HLA were genotyped at high resolution for this locus.. According to the analysis, 78% of the subjects responding against the peptide NS4b_77–91_ were HLA-A*02:01 (Table S3). Subsequently, all the 9-mers covering the NS4b_77–91_ sequence were synthesized and tested for T-cell activation on PBMC from HLA-A*0201 subjects (n = 3 per peptide). For this experiment, the initial 15-mer was used as positive control. Among the 9-mers derived from the NS4b_77–91_, the NS4b_77–85_ (LWNGPMAVS) was the one that induced the most T-cell activation (NS4b_77–91_, used as positive control in Figure 2C). Interestingly, Akondy et al. [24] identified an immunodominant epitope HLA-A*02-restricted on NS4b protein of YF-17D vaccine, the position of which was at amino acid 76–84 (LLWNGPMAV), one amino acid to N-terminal side (Figure 2A). This observation highlights one important caveat of screening peptide libraries, and points at the presence of epitopes not detected due to the manner in which the 15-mer peptides of the library were spliced. Therefore, we tested the adjacent peptide at the position 75–91 for the presence of potential HLA-A*02:01-restricted epitopes within its sequence. Since these peptides have a common core (LWNGPMA), they would represent possible products of antigen processing that could activate the same T cell clone. Thus, to investigate that these peptides could be presented by HLA-A*02:01, CD8^+^ T-cells were isolated from HLA-A*02:01-positive individuals and the ELISPOT assay for IFN-γ was performed using T2 cells, which expresses HLA-A*02:01 exclusively, as target for T-cell activation. Figure 2B depicts representative ELISPOT data set. All peptides analyzed could activate CD8^+^ T-cells, but to different degrees. The peptides with the core LLWNGPMAV (NS4b_76–84_ and NS4b_75–84_) induced the highest number of spots, whereas the peptides in which the core was incomplete (NS4b_77–85_ and NS4b_75–83_) the T-cell response tended to be lower. Finally, we tested the ability of these peptides to expand the population of CD8^+^ T-cells of the patients for one week using aAPC loaded with each of the NS4b peptides. One million purified CD8 T-cells were co-cultured with each aAPC (ratio 1∶1) and after one week the yield of cellular proliferation of the CD8^+^ T-cell population expanded by the aAPCs loaded with NS4b_77–85_, NS4b_76–84_, NS4b_75–83_ or NS4b_75–84_ peptides were 2×10^6^, 4×10^6^, 2.5×10^6^ and 3.5×10^6^ respectively, indicating a proliferation of 2 to 4 fold. Flow cytometry analysis showed that after one week of expansion, epitope-specific CD8^+^ T cells were activated, as degranulating cells (surface CD107a; data not shown) producing IFN-γ (Figure 2C) were identified upon challenging with the same epitopes used during cell expansion. Notably, the expansion of NS4b_76–84_-specific CD8^+^ T-cells was the highest among the peptides tested and reached 22%. Interestingly, the peptide NS4b_75–84_, which on unexpanded CD8^+^ T-cells tested *ex vivo* elicited comparable levels of T-cell response as NS4b_76–84_, had, after one week expansion with the aAPC, a frequency of NS4b_75–84_ specific-CD8^+^ T-cells that was 4-fold less as compared to NS4b_76–84_. These results suggest that there might be different products of antigen processing that could potentially be activating the same T cell clone, however at different degrees. The physiological role of these products of antigen processing for an effective T cell response needs to be further investigated.

### Analysis of HLA Class II Responses

**Figure 3:**
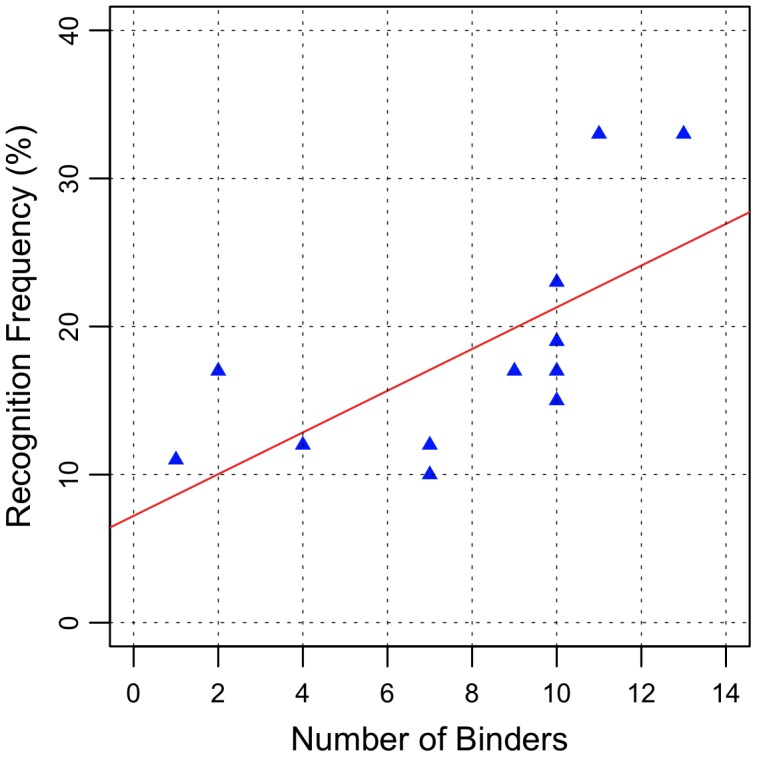
Correlation analysis. The results of ELISPOT and biochemical binding assays indicated that the level of promiscuity of the class II binding was correlated with the prevalence of the immunogenicity of the peptide in the yellow fever cohort.

CD4^+^ and CD8^+^ responses cooperate with each other, enhancing and also regulating T-cell responses. CD4^+^ T-cell helper cytokine responses are required for a proper activation of naïve CD8^+^ T-cells [25] and CD4^+^ helper epitopes linked to each other can also cooperate to allow more efficient T-cell priming of weaker epitopes [26], [27]. Thus, it is reasonable to expect that the most immunogenic peptides could contain both HLA-I and HLA-II binding motifs. Therefore to address this, the binding affinity of the immunogenic peptides was determined for 14 different HLA class II molecules (DRB1*0101, 0301, 0401, 0404, 0405, 0701, 0802, 0901, 1101, 1302, 1501, DRB3*0101, DRB4*0101, DRB5*0101) and the results are shown in Table 6. All the peptides tested could bind with high affinity (IC50≤1000 nM, see [28]) to at least one HLA-DR molecule. Levels of peptide promiscuity varied from low (affinity to 2 to 5 HLA class II molecules bound), e.g. NS5_341–355_ and NS5_345–359_; to intermediate (affinity to 6 to 9 HLA class II molecules bound), e.g. Env_337–351_, NS2b_97–111_ and NS5_465–479_; and highly promiscuous (binding to ≥10 HLA molecules), e.g. Env_57–71_, Env_345–359_, NS4b_77–91_, NS5_469–483_, and NS5_481–495_. Nine peptides (Env_57–71_, Env_345–359_, Env_361–375_, NS3_137–151_, NS4b_77–91_, NS5_341–355_, NS5_345–359_, NS5_465–479_ and NS5_481–495_) contained overlapping HLA-I an HLA-II binding motifs (Tables 3 and 6). This suggested that our screening process targeted the identification of promiscuous T-cell antigens. We then tested whether the frequency of recognition of the peptides on the cohort was dependent on the level of promiscuity of those to different HLA-II molecules. There was a significant correlation (R^2^ = 0.45, p = 0.01) between the number of HLA class II molecules bound to the peptides (Figure 3) and the frequency of positivity in the vaccinees, suggesting that the prevalence of the immunogenicity of an antigen in the population is associated with the HLA promiscuity of the T-cell antigen.

**Table 6:** Binding affinity in nanomols of the most frequent peptides that activated T-cells.

Peptide	DRB1											DRB3	DRB4	DRB5	Total
	*0101	*0301	*0401	*0404	*0405	*0701	*0802	*0901	*1101	*1302	*1501	*0101	*0101	*0101	DR bound
Env_57–71_	**242**	2579	**219**	1104	**459**	**57**	**423**	**457**	**938**	**123**	1846	**197**	**306**	**7.4**	11
Env_337–351_	**31**	**956**	1795	5614	11918	**32**	**846**	**68**	1228	**0.23**	**63**	**119**	1243	**853**	9
Env_345–359_	**41**	**386**	**0.73**	**716**	**189**	**33**	**3.5**	**281**	**24**	**4.2**	**8.1**	2756	**23**	**401**	13
Env_361–375_	**335**	**100**	**7.0**	**911**	**387**	1563	**21**	4314	2574	**30**	**566**	**267**	**578**	6789	10
NS2b_97–111_	**3.9**	19386	3664	4258	**23**	**60**	**222**	**69**	6739	7214	**987**	9903	1183	**64**	7
NS3_137–151_	7356	18002	-	-	-	-	19010	15842	9476	**36**	6310	9292	-	7162	1
NS4b_77–91_	**181**	**84**	**202**	2814	1329	**303**	**348**	**307**	2810	**62**	**575**	**852**	**317**	1281	10
NS5_341–355_	1834	**650**	**201**	-	11772	**20**	1343	11624	-	2270	12683	4835	**478**	9533	4
NS5_345–359_	2837	6737	5694	**319**	**180**	5420	-	-	-	-	9072	3551	4800	7393	2
NS5_465–479_	**72**	5988	**884**	1198	1186	**53**	2388	**428**	4721	2637	**450**	1043	**417**	**537**	7
NS5_469–483_	**26**	5088	2788	**380**	**289**	**247**	**424**	**699**	4183	1919	**202**	**440**	**98**	**513**	10
NS5_481–495_	**30**	**627**	**951**	2898	**73**	**40**	7484	**81**	5925	10577	**299**	**22**	**255**	**80**	10
**Total Peptides bound**	9	6	7	4	7	9	7	8	2	6	8	6	8	7	
**Population Frequency (%)**	9,17	7,33	13,33	-	-	14,33	6,00	1,17	10,67	15,67	13,00	-	-	-	

Numbers in bold: values of IC_50_ below the cutoff of 1000 nmol by which determine that the peptide has affinity to the HLA molecule.

(−): not determined.

## Discussion

This study presents the screening of 653 peptides of the YF-17DD Env, NS1, NS2, NS3, NS4 and NS5 proteins in the context of a cohort of healthy adults immunized with the YF-17DD vaccine. Considering the two rounds of peptide screening, each peptide was tested in at least 75 individuals, and in the case of the Env protein each peptide was tested in 208 volunteers. The screening allowed the identification of 16 T-cell peptides that were immunogenic in 10% or more of the individuals tested. Only a few YF-17D epitopes have been characterized previously [12], [13], [24]. Overall, among the 16 immunogenic peptides identified herein, 14 contain new human T-cell antigens (Env_57–71_, Env_65–79_, Env_73–87_, Env_337–351_, Env_345–359_, Env_361–375_, NS2b_97–111_, NS3_137–151_, NS4a_197–211_, NS5_341–355_, NS5_345–359_, NS5_465–479_, NS5_469–483_, NS5_481–495_). Two peptides, NS2b_113–127_ [12] and the NS4b_77–91_ [24], contained epitopes previously described in humans, while the Env_57–71_ and NS2b_113–127_ peptides contain epitopes that have been described in the murine H2**^d^** background. The murine epitope (Env_57–71_) was reported to be able to stimulate both CD8^+^ and CD4^+^ T-cells to secrete IFN-γ in YF-17DD vaccine immunized H2 **^d^** mice and also HLA A02, B07 and A24 transgenic mice [29], [30]. These highly prevalent immunogenic peptides were shown to contain multiple HLA binding motifs and that the degree of prevalence of its immunogenicity was correlated with the HLA promiscuity. Previous studies have shown some degree of correlation between predicted binding affinity and immunogenicity [30]. However, additional studies are required to determine the precise breath and differences in functionalities of these immunogenic peptides in different HLA contexts.

Biochemical binding assays indicated that the most prevalent immunogenic peptides could bind multiple HLA molecules and the prevalence of their immunogenicity was correlated with the presence of multiple HLA binding motifs. Interestingly, the level of promiscuity of the class II binding was correlated with the prevalence of the immunogenicity of the peptide in the cohort (Figure 2).

No promiscuous T-cell immunogens (peptides that bind more than one HLA allelic variant) have been described for YF wild type virus or other flaviviruses until now. Computational strategies for determination of promiscuous HLA-I and II binding peptides [28], [31] have been used for cancer [32] and infectious disease [33]. Besides the theoretical advantage of being broadly reactive in the population, the biological characteristics of promiscuous T-cell epitopes are not clear and only few promiscuous epitopes have been identified.

CD4^+^ T-cells play a major role in the generation of CD8^+^ cytotoxic T-cell responses and maturation of neutralizing antibodies [34], [35], [36]. In addition, virus-specific CD4^+^ T-cells may be able to tolerate more sequence diversity in their target epitopes than CD8^+^ T-cells, thus being more resistant to mutational escape [37]. Most of the immunogenic peptides, subsequently assessed using competitive binding assays bound to at least six HLA-DR alleles, indicating that an individual bearing at least one such HLA-DR molecule could develop broad CD4^+^ T-cell responses against the YF-17DD. Previous work has shown that many peptides are capable of binding with good affinity to multiple DR alleles [19], [28], [38], [39], [40]. However, it is still not clear how a given promiscuous peptide binds different HLA class II molecules. For example, Kilgus et al. [41] showed that distinct sites on the malaria T-cell epitope interact in different ways with the three DR molecules analyzed [41]. On the other hand, Panina-Bordignon et al. [42] showed that promiscuous peptides interact in a similar way with different DR molecules, possibly by binding to the conserved DR residues [42]. Chicz et al. [43] suggested that the ability of peptides to bind multiple HLA alleles must be dependent on the composition and location of several key amino acids within the primary structure, which led to the hypothesis that rigid allele-specific motifs for the class II molecule do not exist, thus permitting a broad binding specificity [43]. To our knowledge, the present study is the first report showing that the frequency of recognition of the peptides is dependent on the level of promiscuity of those to different HLA molecules. The exact role of the promiscuous peptides in protective immune response still unknown, however one would expect that a vaccine built with multiple immunodominant promiscuous epitopes, capable to bind several HLA molecules, could lead to an increased coverage of the human population. Although the cellular and humoral responses play a central role in effectiveness of YF vaccines, the innate immunity, which is known to shape the development of adaptive immune responses, also contribute to vaccine-mediated protection through other mechanisms, including its live replicative nature and its ability to trigger several Toll-like receptors [6]. In conclusion, the identification of this set of highly prevalent class I and II T-cell epitopes will enable detailed studies of the role of T-cell responses on the development of yellow fever immunity and the identification of the structural requirements of immunogenic promiscuous T-cell epitopes.

## Supporting Information

Table S1 **List of Env and NS peptides sequences that activated T-cells from YF-17DD vaccinees and their responses.**
(DOC)

Table S2 **List of the HLA genotypes of the YF-17DD vaccinees.**
(DOC)

Table S3 **List of HLA genotypes according to immunogenic peptide.**
(DOC)

Box S1 **Description of the Envelope peptide pools.**
(DOC)

Box S2 **Description of the Non-structural peptide pools and matrixes.**
(DOC)
