# Supplementary material for: T-Cell Memory Responses Elicited by Yellow Fever Vaccine are Targeted to Overlapping Epitopes Containing Multiple HLA-I and -II Binding Motifs
Source: PLoS Negl Trop Dis. 2013 Jan 31;7(1):e1938. doi: 10.1371/journal.pntd.0001938 (PMC3561163; doi:10.1371/journal.pntd.0001938)
Supplement: Table S1 — List of Env and NS peptides sequences that activated T-cells from YF-17DD vaccinees and their responses. (DOC) [file pntd.0001938.s001.doc]

**Table S1.** List of Env and NS peptides sequences that activated T-cells from YF-17DD vaccinees and their responses.

| **Peptide** | **Sequence** | **No. tested** | **No. of responders** | **Recognition frequency (%)** | **ELISPOT (SFC/10^6^ PBMC)** | |  |
| --- | --- | --- | --- | --- | --- | --- | --- |
|  |  |  |  |  | **Min** | **Max** | |
| Env_57-71_ | RKVCYNAVLTHVKIN | 30 | 10 | 33 | 25 | 838 | |
| Env_61-75_ | YNAVLTHVKINDKCP | 30 | 2 | 7 | 22 | 197 | |
| Env_65-79_ | LTHVKINDKCPSTGE | 30 | 3 | 10 | 10 | 380 | |
| Env_69-83_ | KINDKCPSTGEAHLA | 30 | 2 | 7 | 45 | 215 | |
| Env_73-87_ | KCPSTGEAHLAEENE | 30 | 3 | 10 | 56 | 323 | |
| Env_217-231_ | WQSGSGGVWREMHHL | 21 | 1 | 5 | 12 | 12 | |
| Env_325-339_ | PKGAPCRIPVIVADD | 18 | 1 | 6 | 20 | 20 | |
| Env_333-347_ | PVIVADDLTAAINKG | 18 | 1 | 6 | 17 | 17 | |
| Env_337-351_ | ADDLTAAINKGILVT | 18 | 3 | 17 | 12 | 30 | |
| Env_341-355_ | TAAINKGILVTVNPI | 27 | 1 | 4 | 6 | 6 | |
| Env_345 -359_ | NKGILVTVNPIASTN | 27 | 9 | 33 | 10 | 271 | |
| Env_353-367_ | NPIASTNDDEVLIEV | 27 | 1 | 4 | 13 | 13 | |
| Env_361-375_ | DEVLIEVNPPFGDSY | 27 | 4 | 15 | 14 | 203 | |
| NS1_57-71_ | LNSVDSLEHEMWRSR | 46 | 1 | 2 | 30 | 30 | |
| NS1_61-75_ | DSLEHEMWRSRADEI | 46 | 1 | 2 | 29 | 29 | |
| NS1_69-83_ | RSRADEINAIFEENE | 46 | 1 | 2 | 23 | 23 | |
| NS1_73-87_ | DEINAIFEENEVDIS | 46 | 4 | 9 | 14 | 45 | |
| NS1_89-103_ | VVQDPKNVYQRGTHP | 46 | 2 | 4 | 22 | 38 | |
| NS1_141-155_ | KECPFSNRVWNSFQI | 46 | 3 | 6 | 20 | 35 | |
| NS1_273-287_ | LEVKREACPGTSVII | 46 | 2 | 4 | 40 | 68 | |
| NS2a_5-19_ | FHEMNNGGDAMYMAL | 59 | 3 | 5 | 20 | 28 | |
| NS2a_9-23_ | NNGGDAMYMALIAAF | 59 | 3 | 5 | 12 | 32 | |
| NS2a_25-39_ | IRPGLLIGFGLRTLW | 59 | 3 | 5 | 11 | 13 | |
| NS2b_5 -19_ | NEALAAAGLVGVLAG | 59 | 2 | 3 | 6 | 30 | |
| NS2b_97 -111_ | VVMTSLALVGAALHP | 59 | 6 | 10 | 10 | 26 | |
| NS2b_101-115_ | SLALVGAALHPFALL | 59 | 1 | 2 | 7 | 42 | |
| NS2b_105-119_* | VGAALHPFALLLVLA | 22 | 2 | 9 | 10 | 13 | |
| NS2b_113-127*_ | ALLLVLAGWLFHVRG | 22 | 3 | 14 | 15 | 181 | |
| NS2b_117-131_ | VLAGWLFHVRGARR | 59 | 2 | 3 | 13 | 27 | |
| NS3_57-71_ | RGAFLVRNGKKLIPS | 46 | 1 | 2 | 12 | 12 | |
| NS3_81-95_ | AYGGSWKLEGRWDGE | 46 | 2 | 4 | 12 | 60 | |
| NS3_117-131_ | SLFKVRNGGEIGAVA | 46 | 2 | 4 | 19 | 90 | |
| NS3_137-151_ | GTSGSPIVNRNGEVI | 46 | 5 | 11 | 18 | 137 | |
| NS3_141-155_ | SPIVNRNGEVIGLYG | 46 | 2 | 4 | 22 | 46 | |
| NS3_181-195_ | ELREIPTMLKKGMTT | 46 | 2 | 4 | 6 | 15 | |
| NS3_205-219_ | KTRRFLPQILAECAR | 46 | 3 | 6 | 10 | 23 | |
| NS3_213-227_ | ILAECARRRLRTLVL | 46 | 2 | 4 | 18 | 63 | |
| NS3_221-235_ | RLRTLVLAPTRVVLS | 46 | 2 | 4 | 10 | 64 | |
| NS3_313-327_ | NESATILMTATPPGT | 46 | 3 | 6 | 18 | 80 | |
| NS3_353-367_ | GHDWILADKRPTAWF | 46 | 1 | 2 | 80 | 80 | |
| NS3_397-411_ | EREYPTIKQKKPDFI | 46 | 2 | 4 | 22 | 26 | |
| NS3_405-419_ | QKKPDFILATDIAEM | 46 | 4 | 9 | 10 | 52 | |
| NS3_409-423_ | DFILATDIAEMGANL | 46 | 3 | 6 | 12 | 59 | |
| NS3_421-435_ | ANLCVERVLDCRTAF | 46 | 4 | 9 | 12 | 46 | |
| NS3_433-447_ | TAFKPVLVDEGRKVA | 46 | 2 | 4 | 10 | 43 | |
| NS3_437-451_ | PVLVDEGRKVAIKGP | 46 | 1 | 2 | 61 | 61 | |
| NS3_453-467_ | RISASSAAQRRGRIG | 46 | 1 | 2 | 12 | 12 | |
| NS4a_9-23_ | LSELPDFLAKKGGEA | 59 | 3 | 5 | 12 | 19 | |
| NS4a_25-39_ | DTISVFLHSEEGSRA | 22 | 2 | 9 | 25 | 74 | |
| NS4a_65-79_ | SGMVIFFMSPKGISR | 22 | 2 | 9 | 58 | 336 | |
| NS4a_69-83_ | IFFMSPKGISRMSMA | 59 | 3 | 5 | 10 | 22 | |
| NS4a_73-87_ | SPKGISRMSMAMGTM | 59 | 2 | 3 | 23 | 36 | |
| NS4a_85-99_ | GTMAGCGYLMFLGGV | 59 | 2 | 3 | 12 | 17 | |
| NS4a_173-187_ | ASPWSWPDLDLKPGA | 59 | 2 | 3 | 11 | 15 | |
| NS4a_177-191_ | SWPDLDLKPGAAWTV | 59 | 2 | 5 | 11 | 34 | |
| NS4a_197-211_ | TMLSPMLHHWIKVEY | 22 | 3 | 14 | 13 | 343 | |
| NS4a_209-223_ | VEYGNLSLSGIAQSA | 59 | 1 | 2 | 11 | 11 | |
| NS4a_253-267_ | ITVMPLLCGIGCAML | 22 | 1 | 4 | 11 | 11 | |
| NS4b_77-91_ | LWNGPMAVSMTGVMR | 59 | 11 | 19 | 17 | 485 | |
| NS4b_97-112_ | FVGVMYNLWKMKTGRR | 59 | 1 | 2 | 11 | 11 | |
| NS5_341-355_ | RMAMTDTTPFGQQRV | 48 | 6 | 12 | 27 | 267 | |
| NS5_345-359_ | TDTTPFGQQRVFKEK | 48 | 8 | 17 | 55 | 914 | |
| NS5_353-367_ | QRVFKEKVDTRAKDP | 48 | 2 | 4 | 12 | 19 | |
| NS5_449-463_ | RTCVYNMMGKREKKL | 48 | 4 | 8 | 10 | 127 | |
| NS5_457-471_ | GKREKKLSEFGKAKG | 48 | 2 | 4 | 11 | 13 | |
| NS5_461-475_ | KKLSEFGKAKGSRAI | 48 | 3 | 6 | 10 | 70 | |
| NS5_465-479_ | EFGKAKGSRAIWYMW | 48 | 6 | 12 | 10 | 1593 | |
| NS5_469-483_ | AKGSRAIWYMWLGAR | 48 | 8 | 17 | 13 | 1828 | |
| NS5_481-495_ | GARYLEFEALGFLNE | 48 | 11 | 23 | 13 | 1022 | |
| NS5_485-499_ | LEFEALGFLNEDHWA | 48 | 3 | 6 | 11 | 43 | |
| NS5_493-507_ | LNEDHWASRENSGGG | 48 | 2 | 4 | 17 | 39 | |
| NS5_609-623_ | YALNTITNLKVQLIR | 48 | 2 | 4 | 262 | 373 | |
| NS5_749-763_ | GWMIKETACLSKAYA | 48 | 4 | 8 | 11 | 33 | |
