# Supplementary material for: T-Cell Memory Responses Elicited by Yellow Fever Vaccine are Targeted to Overlapping Epitopes Containing Multiple HLA-I and -II Binding Motifs
Source: PLoS Negl Trop Dis. 2013 Jan 31;7(1):e1938. doi: 10.1371/journal.pntd.0001938 (PMC3561163; doi:10.1371/journal.pntd.0001938)
Supplement: Table S2 — List of the HLA genotypes of the YF-17DD vaccinees. (DOC) [file pntd.0001938.s002.doc]

**Table S2.** List of the HLA genotypes of the YF-17DD vaccinees.

| **ID Volunteers** | **HLA class I** | | | | | | **HLA class II** | | | | | | |
| --- | --- | --- | --- | --- | --- | --- | --- | --- | --- | --- | --- | --- | --- |
|  | **HLA-A** | | **HLA-B** | | **HLA-C** | | **HLA-DRB1** | | | **HLA-DQB1** | | | |
|  | allele | allele | allele | allele | allele | allele | allele | allele | | allele | | allele | |
| 1 | 32 | 33 | 14 | 49 | 07 | 08 | 07 | | 13 | | 02 | | 06 |
| 2 | 02 | 11 | 44 | 58 | 05 | 07 | 07 | | 13 | | 02 | | 06 |
| 3 | 11 | 23 | 37 | 44 | 06 | 16 | 07 | | 11 | | 02 | | 03 |
| 4 | 23 | 68 | 39 | 41 | 03 | 08 | 08 | | 11 | | 04 | | 06 |
| 5 | 23 | 25 | 18 | 49 | 07 | 12 | 13 | | 15 | | 06 | | 06 |
| 6 | 33 | 68 | 14 | 40 | 03 | 08 | 04 | | 11 | | 03 | | 03 |
| 7 | 01 | 74 | 07 | 81 | 07 | 08 | 12 | | 13 | | 05 | | 06 |
| 8 | 02 | 02 | 48 | 51 | 04 | 14 | 09 | | 11 | | 03 | | 03 |
| 9 | 03 | 23 | 07 | 49 | 07 | 07 | 08 | | 15 | | 03 | | 06 |
| 10 | 01 | 01 | 08 | 51 | 07 | 15 | 04 | | 11 | | 03 | | 03 |
| 11 | 0201 | 24 | 08 | 44 | 05 | 07 | 01 | | 07 | | 02 | | 05 |
| 12 | 0201 | 0201 | 40 | 58 | 03 | 07 | 13 | | 14 | | 05 | | 06 |
| 14 | 24 | 29 | 44 | 58 | 04 | 16 | 07 | | 13 | | 02 | | 06 |
| 15 | 03 | ND | 07 | 44 | 04 | 07 | 04 | | 07 | | 02 | | 02 |
| 16 | 01 | 32 | 50 | 52 | 06 | 12 | 07 | | 15 | | 02 | | 06 |
| 17 | 02 | 03 | 15 | 58 | 03 | 14 | 01 | | 13 | | 05 | | 06 |
| 18 | 01 | 68 | 08 | 49 | 07 | ND | 11 | | 15 | | 03 | | 06 |
| 19 | 03 | 23 | 44 | 49 | 07 | 16 | 07 | | 13 | | 02 | | 03 |
| 20 | 31 | 68 | 15 | 51 | 02 | 15 | 04 | | 11 | | 03 | | 06 |
| 21 | 31 | 68 | 51 | ND | 02 | 15 | 01 | | 13 | | 05 | | 06 |
| 23 | 23 | 26 | 08 | 40 | 03 | 03 | 07 | | 11 | | 02 | | 03 |
| 24 | 03 | 29 | 35 | 51 | 04 | 15 | 08 | | 13 | | 04 | | 06 |
| 25 | 24 | 32 | 40 | 44 | 03 | 05 | 04 | | 08 | | 03 | | 04 |
| 26 | 0201 | 32 | 49 | 53 | 04 | 07 | 13 | | 15 | | 05 | | 06 |
| 27 | 02 | 30 | 27 | 44 | 02 | 16 | 04 | | 15 | | 03 | | 06 |
| 28 | 01 | 02 | 15 | 57 | 07 | 14 | 07 | | 08 | | 03 | | 04 |
| 29 | 0201 | 68 | 38 | 57 | 12 | 12 | 07 | | 13 | | 03 | | 06 |
| 30 | 01 | 03 | 14 | 15 | 03 | 08 | 01 | | 03 | | 02 | | 05 |
| 31 | 29 | 31 | 35 | 42 | 04 | 17 | 03 | | 14 | | 03 | | 04 |
| 32 | 02 | 29 | 53 | 56 | 01 | 04 | 08 | | 15 | | 04 | | 06 |
| 34 | 11 | 24 | 08 | 35 | 04 | 07 | 03 | | 14 | | 02 | | 05 |
| 35 | 02 | 03 | 40 | 50 | 03 | 06 | 07 | | ND | | 02 | | 03 |
| 36 | 01 | 68 | 39 | 40 | 03 | 12 | 04 | | 16 | | 03 | | 05 |
| 37 | 01 | 31 | 08 | 40 | 03 | 07 | 01 | | 04 | | 03 | | 05 |
| 38 | 23 | 32 | 15 | 51 | 14 | 15 | 10 | | 13 | | 05 | | 06 |
| 39 | 11 | 23 | 15 | 44 | 05 | 14 | 01 | | 10 | | 05 | | ND |
| 40 | 03 | 78 | 07 | 51 | 02 | 06 | 07 | | 15 | | 02 | | 06 |
| 41 | 29 | 33 | 44 | 45 | 02 | 07 | 07 | | ND | | 02 | | 02 |
| 42 | 02 | 24 | 15 | 55 | 03 | 03 | 04 | | 14 | | 03 | | 05 |
| 43 | 29 | 68 | ND | ND | ND | ND | ND | | 14 | | 05 | | 06 |
| 44 | 29 | 33 | 14 | 50 | 06 | 08 | 01 | | 08 | | 03 | | 05 |
| 45 | 03 | 29 | 18 | 44 | 02 | 16 | 07 | | 13 | | 02 | | 06 |
| 46 | 24 | 24 | 35 | 35 | 04 | 04 | 04 | | 13 | | 03 | | 06 |
| 47 | 02 | 24 | 15 | 39 | 07 | 14 | 08 | | 14 | | 03 | | 04 |
| 48 | 02 | 29 | 18 | 44 | 07 | 16 | 07 | | 11 | | 02 | | 03 |
| 50 | 11 | 32 | 51 | ND | 14 | 16 | 01 | | 12 | | 03 | | 05 |
| 52 | 02 | 24 | 15 | 51 | 03 | 14 | 08 | | 08 | | ND | | 04 |
| 53 | 0201 | 0201 | 15 | 53 | 03 | 04 | 11 | | 14 | | 03 | | 03 |
| 54 | 29 | 30 | 35 | 44 | 04 | 16 | 07 | | 13 | | 02 | | 06 |
| 55 | 02 | 03 | 07 | 58 | 06 | 07 | 14 | | 15 | | 06 | | 06 |
| 56 | 23 | 30 | 13 | 50 | 06 | ND | 04 | | ND | | 03 | | 03 |
| 57 | 23 | 30 | 13 | 50 | 06 | 06 | 04 | | 11 | | 03 | | 03 |
| 58 | 0201 | 03 | 41 | 49 | 07 | 17 | 04 | | 15 | | 03 | | 06 |
| 59 | 24 | 29 | 35 | 44 | 04 | 16 | 07 | | 15 | | 02 | | 05 |
| 60 | 02 | 03 | 14 | 50 | 06 | 08 | 01 | | 07 | | 02 | | 05 |
| 61 | 02 | 30 | 44 | 57 | 05 | 18 | 04 | | 13 | | 03 | | 05 |
| 64 | 11 | 66 | 51 | 57 | 06 | 16 | 04 | | 12 | | 03 | | 03 |
| 66 | 01 | 02 | 08 | 51 | 04 | 07 | 03 | | 08 | | 02 | | 04 |
| 67 | 01 | 26 | 07 | 52 | 12 | 15 | 15 | | 15 | | 06 | | 06 |
| 68 | 02 | 25 | 14 | 39 | 08 | 12 | 01 | | 16 | | 05 | | ND |
| 69 | 0201 | 68 | 15 | 15 | 03 | 07 | 03 | | 15 | | 02 | | 06 |
| 70 | 24 | 31 | 18 | 82 | 03 | 12 | 11 | | 11 | | 03 | | 03 |
| 71 | 02 | 02 | 15 | 51 | 03 | 16 | 04 | | 11 | | 03 | | 03 |
| 72 | 31 | 68 | 51 | 58 | 07 | 15 | 04 | | 13 | | 03 | | 06 |
| 73 | 02 | 32 | 15 | 51 | 05 | 14 | 04 | | 07 | | 02 | | 03 |
| 74 | 23 | 30 | 15 | 44 | 02 | 03 | 03 | | 13 | | 02 | | 06 |
| 76 | 02 | 33 | 38 | 53 | 04 | 07 | 08 | | 15 | | 03 | | 05 |
| 77 | 02 | 24 | 07 | 45 | 07 | 16 | 0102 | | 15 | | 05 | | 06 |
| 79 | 30 | 33 | 15 | 15 | 03 | 05 | 01 | | 11 | | 03 | | 05 |
| 80 | 31 | 31 | 39 | 39 | 07 | 07 | 04 | | 08 | | 03 | | 04 |
| 81 | 24 | 33 | 18 | 58 | 03 | 07 | 07 | | 13 | | 02 | | 06 |
| 82 | 0201 | 33 | 44 | 58 | 03 | 05 | 11 | | 13 | | 03 | | 06 |
| 84 | 03 | 68 | 35 | 49 | 03 | 12 | 14 | | 15 | | 05 | | 06 |
| 85 | 02 | 26 | 51 | ND | 14 | 16 | 08 | | 13 | | 04 | | 06 |
| 87 | 25 | 30 | 18 | 42 | 12 | 17 | 03 | | 07 | | 02 | | 04 |
| 88 | 0201 | 29 | 44 | 58 | 07 | 16 | 07 | | 08 | | 02 | | 03 |
| 89 | 0201 | 0201 | 44 | 51 | 02 | 05 | 04 | | 07 | | 03 | | 03 |
| 90 | 11 | 26 | 48 | 51 | 04 | 15 | 09 | | 14 | | 03 | | 05 |
| 91 | 01 | 25 | 39 | 44 | 12 | 16 | 07 | | 16 | | 02 | | 05 |
| 92 | 02 | 30 | 40 | 52 | 03 | 15 | 08 | | 16 | | 03 | | 04 |
| 93 | 23 | 68 | 15 | 58 | 02 | 07 | 07 | | 11 | | 02 | | 03 |
| 94 | 02 | 30 | 38 | 57 | 12 | 18 | 13 | | 13 | | 05 | | 06 |
| 95 | 0201 | 0201 | 50 | 51 | 04 | 16 | 07 | | 13 | | 02 | | 03 |
| 96 | 01 | 32 | 40 | 40 | 02 | 15 | 01 | | 14 | | 05 | | 05 |
| 98 | 01 | 24 | 37 | 40 | 02 | 06 | 07 | | 10 | | 03 | | 05 |
| 99 | 03 | 68 | 07 | 15 | 03 | 07 | 12 | | 15 | | 05 | | 06 |
| 100 | 02 | 24 | 07 | 44 | 05 | 07 | 12 | | 13 | | 03 | | 06 |
| 101 | 02 | 74 | 50 | 51 | 06 | 15 | 07 | | 08 | | 02 | | 04 |
| 102 | 0201 | 30 | 13 | 40 | 02 | 06 | 07 | | 11 | | 02 | | 03 |
| 104 | 01 | 24 | 08 | 35 | 04 | 07 | 01 | | 07 | | 03 | | 05 |
| 105 | 11 | 33 | 14 | 55 | 01 | 08 | 01 | | 13 | | 05 | | 06 |
| 106 | 23 | 31 | 15 | 51 | 02 | 14 | 07 | | 14 | | 02 | | 05 |
| 107 | 02 | 26 | 07 | 38 | 07 | 12 | 13 | | 14 | | 05 | | 06 |
| 108 | 03 | 68 | 18 | 51 | 01 | 02 | 07 | | 13 | | 02 | | 02 |
| 110 | 03 | 11 | 08 | 15 | 07 | ND | 03 | | 07 | | 02 | | 03 |
| 113 | 02 | 29 | 18 | 44 | 02 | 05 | 04 | | 04 | | 03 | | ND |
| 114 | 11 | 23 | 44 | 58 | 02 | 07 | 08 | | 13 | | 04 | | 06 |
| 115 | 30 | 34 | 57 | 58 | 06 | 18 | 13 | | 15 | | 05 | | 06 |
| 116 | 02 | 33 | 07 | 14 | 07 | 08 | 01 | | 01 | | 05 | | ND |
| 117 | 02 | 34 | 07 | 57 | 07 | 18 | 01 | | 13 | | 05 | | ND |
| 119 | 23 | 34 | 14 | 53 | 02 | 04 | 01 | | 03 | | 04 | | 05 |
| 120 | 01 | 33 | 08 | 35 | 04 | 07 | 03 | | 04 | | 02 | | 04 |
| 121 | 0201 | 34 | 15 | 52 | 03 | 12 | 04 | | 15 | | 03 | | 06 |
| 123 | 0201 | 03 | 07 | 44 | 04 | 07 | 07 | | 15 | | 02 | | 06 |
| 125 | 0201 | 29 | 07 | 13 | 06 | 07 | 07 | | 15 | | 02 | | 06 |
| 128 | 02 | 24 | 51 | 58 | 07 | 14 | 14 | | ND | | 03 | | 05 |
| 129 | 11 | 24 | 08 | 42 | 07 | 17 | 03 | | 08 | | 02 | | 03 |
| 131 | 02 | ND | 44 | 58 | 03 | 05 | 13 | | 15 | | ND | | 06 |
| 132 | 30 | 80 | 18 | 54 | 02 | 15 | 01 | | 07 | | 02 | | 05 |
| 139 | 0201 | 31 | 44 | 58 | 05 | 07 | 01 | | 10 | | ND | | 05 |
| 151 | 0201 | 32 | 37 | 51 | 06 | 15 | 04 | | 13 | | 03 | | 06 |
| 160 | 0201 | 24 | 15 | 27 | 02 | 03 | 11 | | 16 | | ND | | 03 |
| 161 | 02 | 03 | 35 | 48 | 03 | 04 | ND | | ND | | 03 | | 05 |
| 163 | 01 | 31 | ND | ND | 04 | 15 | 09 | | 13 | | 03 | | 06 |
| 165 | 02 | 26 | 07 | 15 | 03 | 07 | 15 | | 16 | | 05 | | 06 |
| 169 | 0201 | 03 | 18 | 44 | 05 | 12 | 07 | | 13 | | 02 | | 06 |
| 170 | 02 | 23 | 35 | 58 | 04 | 06 | 11 | | 13 | | 03 | | 06 |
| 177 | 24 | ND | 08 | 53 | 04 | 07 | 03 | | 03 | | 02 | | ND |
| 178 | 02 | 23 | 07 | ND | 07 | 17 | 08 | | 15 | | 03 | | 06 |
| 180 | 03 | 03 | 07 | 41 | 07 | 17 | 13 | | 15 | | 06 | | ND |
| 184 | 11 | 68 | 14 | 57 | 06 | 08 | 01 | | 07 | | 03 | | 05 |
| 188 | 02 | ND | 55 | 58 | 03 | 07 | 04 | | 14 | | 03 | | 05 |
| 190 | 30 | 69 | 15 | 15 | 02 | 07 | 09 | | 13 | | 02 | | 06 |
| 191 | 02 | ND | 15 | 44 | 05 | 14 | 01 | | 08 | | 04 | | 05 |
| 192 | 02 | ND | 35 | 40 | 03 | 04 | 04 | | 09 | | 03 | | 03 |
| 194 | 01 | ND | 14 | 51 | 08 | 15 | 07 | | 13 | | 02 | | 06 |
| 200 | 0201 | ND | 15 | 35 | 03 | 04 | 01 | | 16 | | 03 | | 05 |
| 202 | 03 | 26 | 48 | 55 | 03 | 04 | 04 | | 09 | | 03 | | 03 |
| 203 | 30 | 66 | 18 | 39 | 06 | 12 | 03 | | 15 | | 02 | | 06 |
| 204 | 03 | 31 | 54 | 58 | 07 | 07 | 13 | | 15 | | ND | | 06 |
| 208 | 03 | 26 | 37 | 42 | 06 | ND | 10 | | 11 | | 05 | | 06 |
| 212 | 03 | 11 | 38 | 44 | 12 | ND | 07 | | 13 | | 02 | | 06 |
| 214 | 03 | 6801 | 51 | 57 | 12 | 15 | 04 | | 07 | | 03 | | 03 |
| 220 | 26 | 30 | 18 | 35 | 04 | 07 | 13 | | 16 | | 05 | | 06 |
| 221 | 02 | 33 | ND | ND | 04 | 14 | 01 | | 07 | | 02 | | 05 |
| 222 | 24 | 29 | 44 | 45 | 16 | ND | 07 | | 10 | | 02 | | 05 |
| 233 | ND | ND | 18 | 44 | 04 | 12 | 11 | | 13 | | 03 | | 06 |
| 235 | ND | ND | 40 | 52 | 02 | 16 | 13 | | 13 | | 06 | | ND |
| 238 | 30 | 68 | 42 | 49 | 07 | 17 | 03 | | 04 | | 03 | | 04 |
| 242 | 11 | 24 | 15 | 18 | 07 | ND | 03 | | ND | | 02 | | 06 |
| 243 | 01 | 30 | 08 | 73 | 07 | 15 | 03 | | 04 | | 02 | | 03 |
| 246 | 02 | 24 | 39 | 51 | 07 | 14 | 08 | | 14 | | 03 | | ND |

ND: not determined
