# Supplementary material for: T-Cell Memory Responses Elicited by Yellow Fever Vaccine are Targeted to Overlapping Epitopes Containing Multiple HLA-I and -II Binding Motifs
Source: PLoS Negl Trop Dis. 2013 Jan 31;7(1):e1938. doi: 10.1371/journal.pntd.0001938 (PMC3561163; doi:10.1371/journal.pntd.0001938)
Supplement: Table S3 — List of HLA genotypes according to immunogenic peptide. (DOC) [file pntd.0001938.s003.doc]

**Table S3.** List of HLA genotypes according to immunogenic peptide.

| **Peptide** | **ID volunteer** | **HLA class I** | | | | **HLA class II** | | |
| --- | --- | --- | --- | --- | --- | --- | --- | --- |
|  |  | **HLA-A allele** | **HLA-A allele** | **HLA-B allele** | **HLA-B allele** | **HLA-DRB1 allele** | **HLA-DRB1 allele** | |
| Env_57-71_ | 53 | 0201 | 0201 | 15 | 53 | 11 | | 14 |
|  | 58 | 0201 | 03 | 41 | 49 | 04 | | 15 |
|  | 69 | 0201 | 68 | 15 | 15 | 03 | | 15 |
|  | 82 | 0201 | 33 | 44 | 58 | 11 | | 13 |
|  | 88 | 0201 | 29 | 44 | 58 | 01 | | 08 |
|  | 89 | 0201 | 0201 | 44 | 51 | 04 | | 07 |
|  | 95 | 0201 | 0201 | 50 | 51 | 07 | | 13 |
|  | 120 | 01 | 33 | 08 | 35 | 03 | | 04 |
|  | 121 | 0201 | 34 | 15 | 52 | 04 | | 15 |
|  | 214 | 03 | 6801 | 51 | 57 | 04 | | 07 |
| Env_65-79_ | 115 | 30 | 34 | 57 | 58 | 13 | | 15 |
|  | 120 | 01 | 33 | 08 | 35 | 03 | | 04 |
|  | 214 | 03 | 6801 | 51 | 57 | 04 | | 07 |
| Env_73-87*_ | 92 | 02 | 30 | 40 | 52 | 08 | | 16 |
|  | 120 | 01 | 33 | 08 | 35 | 03 | | 04 |
|  | 214 | 03 | 6801 | 51 | 57 | 04 | | 07 |
| Env_337-351_ | 117 | 02 | 34 | 07 | 57 | 01 | | 13 |
|  | 161 | 02 | 03 | 35 | 48 | - | | - |
|  | 163 | 01 | 31 | - | - | 09 | | 13 |
| Env_345-359_ | 11 | 0201 | 24 | 08 | 44 | 01 | | 07 |
|  | 12 | 0201 | 0201 | 40 | 58 | 13 | | 14 |
|  | 29 | 0201 | 68 | 38 | 57 | 07 | | 13 |
|  | 89 | 0201 | 0201 | 44 | 51 | 04 | | 07 |
|  | 102 | 0201 | 30 | 13 | 40 | 07 | | 11 |
|  | 139 | 0201 | 31 | 44 | 58 | 01 | | 10 |
|  | 151 | 0201 | 32 | 37 | 51 | 04 | | 13 |
|  | 160 | 0201 | 24 | 15 | 27 | 11 | | 16 |
|  | 200 | 0201 | - | 15 | 35 | 01 | | 16 |
| Env_361-375_ | 5 | 23 | 25 | 18 | 49 | 13 | | 15 |
|  | 90 | 11 | 26 | 48 | 51 | 09 | | 14 |
|  | 132 | 30 | 80 | 18 | 54 | 01 | | 07 |
|  | 220 | 26 | 30 | 18 | 35 | 13 | | 16 |
| NS2b_97 -111_ | 12 | 0201 | 0201 | 40 | 58 | 13 | | 14 |
|  | 16 | 01 | 32 | 50 | 52 | 07 | | 15 |
|  | 32 | 02 | 29 | 53 | 56 | 08 | | 15 |
|  | 72 | 31 | 68 | 51 | 58 | 04 | | 13 |
|  | 180 | 03 | 03 | 07 | 41 | 13 | | 15 |
|  | 190 | 30 | 69 | 15 | 15 | 09 | | 13 |
| NS2b_113 -127_* | 115 | 30 | 34 | 57 | 58 | 13 | | 15 |
|  | 120 | 01 | 33 | 08 | 35 | 03 | | 04 |
|  | 214 | 03 | 6801 | 51 | 57 | 04 | | 07 |
| NS3_137-151_ | 50 | 11 | 32 | 51 | - | 01 | | 12 |
|  | 105 | 11 | 33 | 14 | 55 | 01 | | 13 |
|  | 170 | 02 | 23 | 35 | 58 | 11 | | 13 |
|  | 221 | 02 | 33 | - | - | 01 | | 07 |
|  | 242 | 11 | 24 | 15 | 18 | 03 | | - |
| NS4a_197-211_* | 92 | 02 | 30 | 40 | 52 | 08 | | 16 |
|  | 120 | 01 | 33 | 08 | 35 | 03 | | 04 |
|  | 214 | 03 | 6801 | 51 | 57 | 04 | | 07 |
| NS4b_77-91_ | 12 | 0201 | 0201 | 40 | 58 | 13 | | 14 |
|  | 19 | 03 | 23 | 44 | 49 | 07 | | 13 |
|  | 26 | 0201 | 32 | 49 | 53 | 13 | | 15 |
|  | 29 | 0201 | 68 | 38 | 57 | 07 | | 13 |
|  | 95 | 0201 | 0201 | 50 | 51 | 07 | | 13 |
|  | 123 | 0201 | 03 | 07 | 44 | 07 | | 15 |
|  | 125 | 0201 | 29 | 07 | 13 | 07 | | 15 |
|  | 169 | 0201 | 03 | 18 | 44 | 07 | | 13 |
|  | 184 | 11 | 68 | 14 | 57 | 01 | | 07 |
| NS5_341-355_ | 4 | 23 | 68 | 39 | 41 | 08 | | 11 |
|  | 28 | 01 | 02 | 15 | 57 | 07 | | 08 |
|  | 36 | 01 | 68 | 39 | 40 | 04 | | 16 |
|  | 121 | 02 | 34 | 15 | 52 | 04 | | 15 |
|  | 203 | 30 | 66 | 18 | 39 | 03 | | 15 |
|  | 214 | 03 | 6801 | 51 | 57 | 04 | | 07 |
| NS5_345-359_ | 4 | 23 | 68 | 39 | 41 | 08 | | 11 |
|  | 24 | 03 | 29 | 35 | 51 | 08 | | 13 |
|  | 36 | 01 | 68 | 39 | 40 | 04 | | 16 |
|  | 54 | 29 | 30 | 35 | 44 | 07 | | 13 |
|  | 120 | 01 | 33 | 08 | 35 | 03 | | 04 |
|  | 203 | 30 | 66 | 18 | 39 | 03 | | 15 |
|  | 208 | 03 | 31 | 54 | 58 | 13 | | 15 |
|  | 214 | 03 | 6801 | 51 | 57 | 04 | | 07 |
| NS5_465-479_ | 20 | 31 | 68 | 15 | 51 | 04 | | 11 |
|  | 26 | 02 | 32 | 49 | 53 | 13 | | 15 |
|  | 28 | 01 | 02 | 15 | 57 | 07 | | 08 |
|  | 38 | 23 | 32 | 15 | 51 | 10 | | 13 |
|  | 43 | 29 | 68 | - | - | 14 | | - |
|  | 55 | 02 | 03 | 07 | 58 | 14 | | 15 |
| NS5_469-483_ | 26 | 02 | 32 | 49 | 53 | 13 | | 15 |
|  | 28 | 01 | 02 | 15 | 57 | 07 | | 08 |
|  | 55 | 02 | 03 | 07 | 58 | 14 | | 15 |
|  | 128 | 02 | 24 | 51 | 58 | 14 | | - |
|  | 202 | 02 | 26 | 48 | 55 | 04 | | 09 |
|  | 214 | 03 | 6801 | 51 | 57 | 04 | | 07 |
| NS5_481-495_ | 3 | 11 | 23 | 37 | 44 | 07 | | 11 |
|  | 4 | 23 | 68 | 39 | 41 | 08 | | 11 |
|  | 5 | 23 | 25 | 18 | 49 | 13 | | 15 |
|  | 19 | 03 | 23 | 44 | 49 | 07 | | 13 |
|  | 23 | 23 | 26 | 08 | 40 | 07 | | 11 |
|  | 25 | 24 | 32 | 40 | 44 | 06 | | 08 |
|  | 38 | 23 | 32 | 15 | 51 | 10 | | 13 |
|  | 39 | 11 | 23 | 15 | 44 | 01 | | 10 |
|  | 52 | 02 | 24 | 15 | 51 | 08 | | 08 |
|  | 56 | 23 | 30 | 13 | 50 | 04 | | - |
|  | 214 | 03 | 6801 | 51 | 57 | 04 | | 07 |

The Table shows the HLA genotyping of subjects that responded to the most immunogenic 15-mer peptides.

*Peptides not analyzed by binding assay
