# Supplementary material for: T-Cell Memory Responses Elicited by Yellow Fever Vaccine are Targeted to Overlapping Epitopes Containing Multiple HLA-I and -II Binding Motifs
Source: PLoS Negl Trop Dis. 2013 Jan 31;7(1):e1938. doi: 10.1371/journal.pntd.0001938 (PMC3561163; doi:10.1371/journal.pntd.0001938)
Supplement: Box S1 — Description of the Envelope peptide pools. (DOC) [file pntd.0001938.s004.doc]

**Box S1**: Description of the Envelope peptide pools.

| **Envelope peptides** | | | | | | |
| --- | --- | --- | --- | --- | --- | --- |
| **POOL** |  |  |  |  |  |  |
| **1** | 1-15 | 5-19 | 9-23 | 13-27 | 17-31 | 21-35 |
| **2** | 25-39 | 29-43 | 33-47 | 41-55 | 45-59 | 49-63 |
| **3** | 53-67 | 57-71 | 61-75 | 65-79 | 69-83 | 73-87 |
| **4** | 77-91 | 81-95 | 85-99 | 89-103 | 93-107 | 97-111 |
| **5** | 101-115 | 105-119 | 109-123 | 113-127 | 117-131 | 121-135 |
| **6** | 125-139 | 129-143 | 133-147 | 137-151 | 141-155 | 145-159 |
| **7** | 149-163 | 153-167 | 157-171 | 161-175 | 165-179 | 169-183 |
| **8** | 173-187 | 177-191 | 181-195 | 185-199 | 189-203 | 193-207 |
| **9** | 197-211 | 201-215 | 205-219 | 209-223 | 213-227 | 217-231 |
| **10** | 221-235 | 225-239 | 229-243 | 233-247 | 237-251 | 241-255 |
| **11** | 245-259 | 249-263 | 253-267 | 257-271 | 261-275 | 265-279 |
| **12** | 269-283 | 273-287 | 277-291 | 281-295 | 285-299 | 289-303 |
| **13** | 293-307 | 297-311 | 301-315 | 305-319 | 309-323 | 313-327 |
| **14** | 317-331 | 321-335 | 325-339 | 329-343 | 333-347 | 337-351 |
| **15** | 341-355 | 345-359 | 349-363 | 353-367 | 357-371 | 361-375 |
| **16** | 365-379 | 369-383 | 373-387 | 377-391 | 381-395 | 385-399 |
| **17** | 389-403 | 393-407 | 397-409 | 401-415 | 405-419 | 409-423 |
| **18** | 413-427 | 417-431 | 421-435 | 425-439 | 429-443 | 433-447 |
| **19** | 437-451 | 441-455 | 445-459 | 449-463 | 453-467 | 457-471 |
| **20** | 461-475 | 465-479 | 469-483 | 473-487 | 477-491 | 481-493 |
