# Supplementary material for: T-Cell Memory Responses Elicited by Yellow Fever Vaccine are Targeted to Overlapping Epitopes Containing Multiple HLA-I and -II Binding Motifs
Source: PLoS Negl Trop Dis. 2013 Jan 31;7(1):e1938. doi: 10.1371/journal.pntd.0001938 (PMC3561163; doi:10.1371/journal.pntd.0001938)
Supplement: Box S2 — Description of the Non-structural peptide pools and matrixes. (DOC) [file pntd.0001938.s005.doc]

**Box S2**: Description of the Non-structural peptide pools and matrixes.

| **NS1 peptides** | | | | | | | |
| --- | --- | --- | --- | --- | --- | --- | --- |
| **POOL** | **1** | **2** | **3** | **4** | **5** | **6** | **7** |
| **8** | 1-15 | 21-35 | 25-39 | 29-43 | 33-47 | 37-51 | 49-63 |
| **9** | 57-71 | 61-75 | 69-83 | 73-87 | 77-91 | 85-99 | 89-103 |
| **10** | 93-107 | 97-111 | 105-119 | 109-123 | 117-131 | 121-135 | 129-143 |
| **11** | 133-147 | 141-155 | 181-195 | 185-199 | 193-207 | 205-219 | 213-227 |
| **12** | 217-231 | 221-235 | 237-251 | 245-259 | 249-263 | 253-267 | 265-279 |
| **13** | 269-283 | 273-287 | 281-295 | 293-307 | 333-347 | 337-351 | 341-355 |
| **14** | 361-375 | 385-399 | 389-403 | 393-407 | 397-409 |  |  |

| **NS2a peptides** | | | | | | | | |
| --- | --- | --- | --- | --- | --- | --- | --- | --- |
| **POOL** | **1** | **2** | **3** | **4** | **5** | **6** | **7** | **8** |
| **9** | 1-15 | 5-19 | 9-23 | 21-35 | 25-39 | 33-47 | 37-51 | 41-55 |
| **10** | 49-63 | 57-71 | 61-75 | 65-79 | 69-83 | 73-87 | 77-91 | 81-95 |
| **11** | 85-99 | 89-103 | 93-107 | 97-111 | 105-119 | 109-123 | 113-127 | 117-131 |
| **12** | 121-135 | 129-143 | 133-147 | 137-151 | 141-155 | 145-159 | 149-163 | 153-167 |

| **NS2b peptides** | | | | | | | |
| --- | --- | --- | --- | --- | --- | --- | --- |
| **POOL** | **1** | **2** | **3** | **4** | **5** | **6** | **7** |
| **8** | 1-15 | 5-19 | 13-27 | 17-31 | 21-35 | 25-39 | 29-43 |
| **9** | 33-47 | 37-51 | 41-55 | 45-59 | 49-63 | 53-67 | 57-71 |
| **10** | 61-75 | 65-79 | 69-83 | 73-87 | 77-91 | 81-95 | 85-99 |
| **11** | 89-103 | 97-111 | 101-115 | 109-123 | 117-131 |  |  |

| **NS4a peptides** | | | | | | | | |
| --- | --- | --- | --- | --- | --- | --- | --- | --- |
| **POOL** | **1** | **2** | **3** | **4** | **5** | **6** | **7** | **8** |
| **9** | 1-15 | 5-19 | 9-23 | 13-27 | 17-31 | 29-43 | 33-47 | 37-51 |
| **10** | 41-55 | 69-83 | 73-87 | 85-99 | 89-103 | 93-107 | 97-111 | 113-127 |
| **11** | 117-131 | 121-135 | 129-143 | 149-163 | 153-167 | 157-171 | 161-175 | 165-179 |
| **12** | 169-183 | 173-187 | 177-191 | 181-195 | 193-207 | 201-215 | 205-219 | 209-223 |
| **13** | 221-235 | 229-243 | 261-275 | 265-279 | 269-283 | 269-283 | 273-287 |  |

| **NS4b peptides** | | | | |
| --- | --- | --- | --- | --- |
| **POOL** | **1** | **2** | **3** | **4** |
| **5** | 1-15 | 5-19 | 9-23 | 13-27 |
| **6** | 17-31 | 21-35 | 37-51 | 45-59 |
| **7** | 53-67 | 57-71 | 61-75 | 65-79 |
| **8** | 77-91 | 81-95 | 85-99 | 97-112 |

| **NS3 peptides** | | | | | | | | | | | | | | | |
| --- | --- | --- | --- | --- | --- | --- | --- | --- | --- | --- | --- | --- | --- | --- | --- |
| **POOL** | **1** | **2** | **3** | **4** | **5** | **6** | **7** | **8** | **9** | **10** | **11** | **12** | **13** | **14** | **15** |
| **16** | 1-15 | 5-19 | 9-23 | 13-27 | 17-31 | 21-35 | 25-39 | 29-43 | 33-47 | 37-51 | 41-55 | 45-59 | 49-63 | 53-67 | 57-71 |
| **17** | 61-75 | 65-79 | 69-83 | 73-87 | 77-91 | 81-95 | 85-99 | 89-103 | 93-107 | 97-111 | 101-115 | 105-119 | 109-123 | 113-127 | 117-131 |
| **18** | 121-135 | 125-139 | 129-143 | 133-147 | 137-151 | 141-155 | 145-159 | 149-163 | 161-175 | 165-179 | 169-183 | 173-187 | 177-191 | 181-195 | 189-203 |
| **19** | 193-207 | 197-211 | 201-215 | 205-219 | 209-223 | 213-227 | 217-231 | 221-235 | 225-239 | 229-243 | 233-247 | 237-251 | 241-255 | 245-259 | 249-263 |
| **20** | 253-267 | 257-271 | 261-275 | 265-279 | 269-283 | 273-287 | 277-291 | 285-299 | 289-303 | 293-307 | 297-311 | 301-315 | 305-319 | 309-323 | 313-327 |
| **21** | 317-331 | 321-335 | 325-339 | 329-343 | 333-347 | 337-351 | 341-355 | 345-359 | 349-361 | 353-367 | 357-371 | 361-375 | 365-379 | 369-383 | 373-387 |
| **22** | 377-391 | 381-395 | 385-399 | 389-403 | 393-407 | 397-411 | 401-415 | 405-419 | 409-423 | 413-427 | 417-431 | 421-435 | 425-439 | 429-443 | 433-447 |
| **23** | 437-451 | 441-455 | 445-459 | 449-463 | 453-467 | 457-471 | 461-475 | 465-479 | 469-483 | 473-487 | 477-491 | 481-495 | 485-499 | 489-503 | 493-507 |
| **24** | 497-511 | 501-515 | 505-519 | 509-523 | 513-527 | 517-531 | 521-535 | 525-539 | 529-543 | 533-547 | 537-551 | 541-555 | 545-559 | 549-563 | 553-567 |
| **25** | 557-571 | 561-575 | 565-579 | 569-583 | 573-587 | 577-591 | 581-595 | 585-599 | 589-603 | 593-607 | 597-611 | 601-615 | 609-624 |  |  |

| **NS5 peptides** | | | | | | | | | | | | | | | | | |
| --- | --- | --- | --- | --- | --- | --- | --- | --- | --- | --- | --- | --- | --- | --- | --- | --- | --- |
| **POOL** | **1** | **2** | **3** | **4** | **5** | **6** | **7** | **8** | **9** | **10** | **11** | **12** | **13** | **14** | **15** | **16** | **17** |
| **18** | 1-15 | 5-19 | 9-23 | 13-27 | 17-31 | 21-35 | 25-39 | 29-43 | 33-47 | 37-51 | 41-55 | 45-59 | 49-63 | 53-67 | 57-71 | 61-75 | 65-79 |
| **19** | 69-83 | 73-87 | 77-91 | 81-95 | 85-99 | 89-103 | 93-107 | 97-111 | 101-115 | 105-119 | 109-123 | 113-127 | 117-131 | 121-135 | 129-143 | 133-147 | 137-151 |
| **20** | 141-155 | 145-159 | 153-167 | 157-171 | 161-175 | 165-179 | 169-183 | 173-187 | 177-191 | 181-195 | 185-199 | 189-203 | 193-207 | 197-211 | 201-215 | 205-219 | 209-223 |
| **21** | 213-227 | 217-231 | 221-235 | 225-239 | 229-243 | 233-247 | 237-251 | 241-255 | 249-263 | 253-267 | 257-271 | 261-275 | 265-279 | 269-283 | 273-287 | 277-291 | 281-295 |
| **22** | 285-299 | 289-303 | 293-307 | 297-311 | 301-315 | 305-319 | 313-327 | 317-331 | 321-335 | 325-339 | 329-343 | 333-347 | 337-351 | 341-355 | 345-359 | 349-361 | 353-367 |
| **23** | 357-371 | 361-375 | 365-379 | 369-383 | 373-387 | 377-391 | 381-395 | 385-399 | 389-403 | 393-407 | 397-411 | 401-415 | 405-419 | 409-423 | 413-427 | 417-431 | 421-435 |
| **24** | 425-439 | 429-443 | 433-447 | 437-451 | 441-455 | 445-459 | 449-463 | 453-467 | 457-471 | 461-475 | 465-479 | 469-483 | 477-491 | 481-495 | 485-499 | 493-507 | 497-511 |
| **25** | 501-515 | 505-519 | 509-523 | 513-527 | 517-531 | 521-535 | 525-539 | 529-543 | 533-547 | 537-551 | 541-555 | 545-559 | 549-563 | 553-567 | 557-571 | 561-575 | 565-579 |
| **26** | 569-583 | 573-587 | 577-591 | 581-595 | 585-599 | 589-603 | 593-607 | 597-611 | 601-615 | 605-619 | 609-623 | 613-627 | 621-635 | 625-639 | 629-643 | 633-647 | 637-651 |
| **27** | 641-655 | 645-659 | 649-663 | 653-667 | 657-671 | 661-675 | 665-679 | 669-683 | 673-687 | 677-691 | 681-695 | 685-699 | 689-703 | 693-707 | 697-711 | 701-715 | 705-719 |
| **28** | 709-723 | 713-727 | 717-731 | 721-735 | 725-739 | 729-743 | 733-747 | 737-751 | 741-755 | 745-759 | 749-763 | 753-767 | 757-771 | 761-775 | 765-779 | 769-783 | 773-787 |
| **29** | 777-791 | 781-795 | 785-799 | 789-803 | 793-807 | 801-815 | 805-819 | 809-823 | 813-827 | 817-831 | 821-835 | 825-839 | 829-843 | 833-847 | 837-851 | 841-855 | 845-859 |
| **30** | 849-863 | 853-867 | 857-871 | 861-875 | 865-879 | 869-883 | 873-887 | 877-891 | 881-895 | 885-899 | 889-905 | 489-503 |  |  |  |  |  |
